# Genome-wide identification of geographical segregated genetic markers in *Salmonella enterica* serovar Typhimurium variant 4,[5],12:i:-

**DOI:** 10.1038/s41598-018-33266-5

**Published:** 2018-10-15

**Authors:** Federica Palma, Gerardo Manfreda, Mickael Silva, Antonio Parisi, Dillon O. R. Barker, Eduardo N. Taboada, Frédérique Pasquali, Mirko Rossi

**Affiliations:** 1 Department of Agricultural and Food Sciences, School of Agriculture and Veterinary Medicine, University of Bologna, Bologna, Italy; 2 Instituto de Microbiologia, Instituto de Medicina Molecular, Faculdade de Medicina, Universidade de Lisboa, Lisbon, Portugal; 3 Istituto Zooprofilattico Sperimentale della Puglia e della Basilicata, Foggia, Italy; 4 National Microbiology Laboratory at Lethbridge, Public Health Agency of Canada, Lethbridge, Canada; 5 Department of Food Hygiene and Environmental Health, Faculty of Veterinary Medicine, University of Helsinki, Helsinki, Finland

## Abstract

*Salmonella enterica* ser. Typhimurium monophasic variant 4,[5],12:i:- has been associated with food-borne epidemics worldwide and swine appeared to be the main reservoir in most of the countries of isolation. However, the monomorphic nature of this serovar has, so far, hindered identification of the source due to expansion of clonal lineages in multiple hosts and food producing systems. Since geographically structured genetic signals can shape bacterial populations, identification of biogeographical markers in *S*. 1,4,[5],12:i:- genomes can contribute to improving source attribution. In this study, the phylogeographical structure of 148 geographically and temporally related Italian *S*. 1,4,[5],12:i:- has been investigated. The Italian isolates belong to a large population of clonal *S*. Typhimurium/1,4,[5],12:i:- isolates collected worldwide in two decades showing up to 2.5% of allele differences. Phylogenetic reconstruction revealed that isolates from the same geographical origin form highly supported monophyletic groups, suggesting discrete geographical segregation. These monophyletic groups are characterized by the gene content of a large *sopE*-containing prophage. Within this prophage, genome-wide comparison identified several genes overrepresented in strains of Italian origin. This suggests that certain lineages may be characterized by the acquisition of specific accessory genetic markers useful for improving identification of the source in ongoing epidemics.

## Introduction

*Salmonella enterica* servovar Typhimurium with the antigenic formula 4,[5],12:i:- is considered a monophasic variant of *S*. Typhimurium (MVSTm) lacking the second phase flagellar antigen^1^. MVSTm has recently emerged in food-borne epidemics of multi-drug resistance (MDR) strains responsible for several outbreaks in Europe (EU)^2^ as well as in other continents^1^. Since this serovar was detected, as far back in 1997^3^, it has been repeatedly associated to humans and swine production, but also to environmental samples and other food-producing animals, such as avian and cattle^2,4–8^. The increasing spread of MVSTm in EU, the growing number of food-borne outbreaks in recent years^6^ and the difficulties in identifying the source due to the monomorphic nature of this serovar continue to be a public health concerns. The existence of at least two distinct clones (European and Spanish clone) emerging independently from ancestral *S*. Typhimurium strains has been previously described^2,5,9–11^. Additionally, different antimicrobial resistance (AR) patterns have been associated to both clones. The prevalence of simultaneous resistance to ampicillin, streptomycin/spectinomycin, sulphonamides and tetracycline (R-type ASSuT) has been described in strains from EU clone^12^, while in strains from the Spanish clone an additional resistance to chloramphenicol, gentamycin and trimethoprim has been reported^13^.

It has been argued that traditional typing methods are not well suited to unravel the evolution dynamics of MVSTm population, as well as the source attribution and epidemiology of this monomorphic bacterial pathogen^14^. Moreover, the misclassification of this serovar due to the technically-demanding serotyping protocols and the evolution of multiple monophasic genotypes, make tackling the phylogenetic differentiation of MVSTm from serovar Typhimurium more challenging^15,16^. On the other hand, large-scale genomic approaches based on core genome multi-locus sequence typing (cgMLST) and single nucleotide polymorphisms (SNPs) phylogenies have shown an invaluable potential for WGS- subtyping of genetically closely related strain. Additionally, the constant growing of public database containing *Salmonella* genomes (currently including >100,000) represent an unforeseeable opportunity to analyse population structure or epidemiological transmission chain within single populations^17,18^. Recent studies have shown that combining core genome analysis with accessory genes pool analysis, such as pan-genome wide association studies (pan-GWAS), has improved understanding on evolutionary and phylogeographic patterns of several food-borne bacterial pathogens^19–22^.

Geographical structure of bacterial population is well documented for several pathogens such as *Mycobacterium tuberculosis*^23^ and *Helicobacter pylori*^24^. However, for food-borne pathogens^25–28^ the local phylogeographical signals quickly deteriorate by the rapid movement of lineages across the globe due to international trade of food and animals, and human travel^25,29^. Nevertheless, the adaptation of certain lineages to specific hosts or food production systems, which are more relevant on a local geographical scale, may result in the expansion of successful epidemic clones harbouring unique gene clusters. These clusters constitute specific biomarkers that may be used to improve source attribution in strains circulating in different countries. Therefore, in this study the phylogeographical structure of a set of 148 geographically and temporally related MVSTm isolates collected in Italy between 2012 and 2014 from human and swine was investigated in an extended context of selected publicly available *S*. Typhimurium/MVSTm strains from several countries.

Combining phylogenetic analysis and genome-wide association study (GWAS) we found strong evidence of the phylogeographical structure of the MVSTm isolates, identifying a specific SopEϕ-like phage as biomarker for a clone that has recently spread widely in Italy.

## Results

### Quality of *de novo* assembly

All the draft genome sequences from 148 Italian (herein STY) MVSTm isolates originating from human and swine passed the QA/QC measures as defined in INNUca pipeline (https://github.com/theInnuendoProject/INNUca). *In silico* MLST classified 142 (96%) samples as Sequence Type (ST)-34, 3 (2%) as ST-19, 1 (0.7%) as ST-11 and 1 (0.7%) as ST-1995. For one isolate MLST ST was not found.

### Population structure *Salmonella* genomes

Population structure analysis has been performed based on a core genome gene-by-gene approach using the chewBBACA^30^ suite (https://github.com/B-UMMI/chewBBACA), to untangle the geospatial evolution of the 148 Italian STY MVSTm isolates in the context of a representative set (4,312) of publicly available *Salmonella enterica* genomes including the most common serovars. A total of 3,255 out of the 8,558 loci in the wgMLST schema have been detected in >99% of the samples and used for the cgMLST study. No concordance has been found between serotyping and any goeBURST clusters based on the 3,255 loci cgMLST schema (Adjusted Wallace Coefficient (AWC) <0.6). However, considering serovar 1,4,[5],12:i:- as Typhimurium, concordance between cgMLST clustering and serotyping have been found at ~30% (965) of allele differences (bidirectional AWC >0.97), including 35 goeBURST groups. At 965 cut-off, 141 out of 148 STY MVSTm isolates belong to a single group along with 2,595 genomes including the majority of *S*. Typhimurium and other MVSTm publicly available strains. Besides, a large part of the STY MVSTm isolates (136 out of 148; ~92%) cluster in a single goeBURST group at ~2.5% (75) of allele differences (referred as “goeBURST^75^”) along with a mixed population of 241 *S*. Typhimurium and 912 MVSTm publicly available genomes (Supplementary Table S1). The MVSTm strains were isolated between 2001 and 2017 from human (52%), swine (14%) and other sources (22%) (124 environment, 50 avian and 24 cattle). For 111 isolates no source of isolation was available. Almost the 94% of these strains were collected in Western Europe (433) and North America (427) while the remaining isolates were from Northern Europe (35), Southern Europe (10), Asia (4) and Eastern Europe (1). Publicly available MVSTm belong to ST-34 (870), ST-19 (25) ST-2379 (12), ST-2956 (1), ST-3168 (1) and ST-3224 (1). Most *S*. Typhimurium genomes (227) were classified as ST-34, of which more than half (115) were human isolates mainly from North America and Western Europe. For goeBURST^75^, the Minimum Spanning Tree (MST) based on a new cgMLST schema, including a total of 3,591 loci, has been calculated using Phyloviz 2.0^31^ and the country of origin of the strains have been visualized on the tree (Supplementary Fig. S1). Figure S1 showed a partial enrichment of geographical linked strains in certain parts of the MST (e.g. Italian cluster, yellow circle).

### Pangenome analysis

Pangenome analysis has been performed using Roary^32^ on a total of 1,326 genomes comprising all the 1,289 genomes belonging to goeBURST^75^ (including the 136 STY MVSTm isolates) and an outgroup composed by a set of 38 *S*. Typhimurium/MVSTm strains, including 27 Italian MVSTm, 19 of which isolated during a large outbreak in Southern Italy^33^. The pangenome consists of a matrix of 13,135 group of orthologues of which 9,085 are accessory (present in <99% of genomes): 8,588 present in less than 15% of strains, 333 present in more than 15% but less than 95% of strains, and 164 genes present in more than 95% of strains but less than 99%. A distance tree was inferred based on binary data of presence/absence of accessory gene using IQtree^34^ (Supplementary Fig. S2). Two major clusters have been identified: (a) including 1,233 strains mainly of ST-34, and (b) containing 98 strains mainly of ST-19.

Within the large ST-34 cluster, *S*. Typhimurium/MVSTm strains are aggregated in clades irrespectively of the year as well as of the source of isolation (Fig. S2). However, even if isolates originating from different countries are gathered together across big clades, several small clusters including isolates of the same geographical area could be visually identified across the tree. In particular, the clade named herein STY-clade (Fig. S2) is populated by 98 isolates of which roughly 71% (70) were Italian while the remainder were from Western Europe (24) and North America (4). Isolates from this clade were collected between 2007 and 2017 and obtained from human, swine, and cattle. These data suggest that certain lineages might contain specific accessory genetic markers as a result of geographical segregation.

### *In silico* characterization of the isolates

A comprehensive list of the plasmids detected in the 1,326 genomes is available in Supplementary Table S2. The presence of plasmids has been detected in most of the genomes (1214; 91.5%) based on the positive match against PlasmidFinder database^35^. The most frequently reported plasmid was IncQ1 (959; 72.3%) followed by ColRNAI (506; 38.2%), Col156 (244; 18.4%). In total, the simultaneous presence of these three plasmids was observed in 77 ST-34 strains of which ~48% (37) clusters within the above described STY-clade. Overall, less than 6% of isolates were positive for further plasmid including several incompatibility groups Inc. Although none of the tested strains harboured the complete 94 kb virulent pSLT plasmid, remains of the plasmid have been detected exclusively in the ST-19 clade which includes 21 MVSTm isolates possessing from 4 to 32 of the pSLT coding DNA sequences (CDSs), comprising the virulent markers *spvB* and *spvC*^36^. All the MVSTm outbreak isolates characterized by Cito and colleagues^33^ and 2 out 6 STY MVSTm isolates clustered within ST-19 clade do not possess any pSLT CDSs. In contrast, the other 4 STY MVSTm within ST-19 clade isolates possess from 4 to 30 pSLT CDSs.

Almost all genomes (1,279; 96.4%) are positive for at least one antimicrobial resistance associated gene (ARG) and most of them (1,011; 76.2%) possess three or more ARGs, while a limited number (3.6%) did not have any positive match in Resfinder database^37^ (Supplementary Table S3). The most prevalent ARGs were related to resistance to tetracycline (89.7%), sulphonamides (76.6%), ampicillin (74%), streptomycin (75.6%). More specifically, the simultaneous presence of genes for resistance to ampicillin (*bla*_*TEM-1B*_), streptomycin (*strA*, *strB*, or *aph(3“)-Ib*, *aph(6)-Id*), sulphonamide (*sul1* and *sul2*) and tetracycline (*tet(A)* or *tet(B)*) predicting the ASSuT resistotype (R-type) and characterizing the so called European clone have been found in 67% of the positive isolates which were originated from human and swine sources and collected between 2004 and 2017. Particularly, a total of 105 over 167 isolates of Italian origin (~83% of the genomes within the STY-clade) exhibit R-type ASSuT. Only 18 over 1,326 genomes were classified as R-type ASSuTCGTp due to the simultaneous presence of additional genes for resistance to gentamycin (*aac(3)-IVa*), trimethoprim (*dfrA12*), and chloramphenicol (*cmlA1*). These isolates, predicted to harbour the R-type typical of MVSTm described as part of the Spanish clone, were collected from human sources and interspersed among clusters including isolates from different sources and with R-type ASSuT.

Colistin resistance related genes *mcr-1*, *mcr-3*, *mcr-4* or *mcr-5* were revealed in only 10 of the ST-34 genomes mainly classified as MVSTm (8/10), collected in Italy, UK and Thailand from swine (5/10) and human. None of the genomes belonging to the STY-clade are positive for colistin resistance genes.

### Genome-Wide Association Study identified genetic markers in Italian MVSTm

To investigate which genetic traits may be associated with specific MVSTm genotype that has shown a successful local expansion in Italy, we used Scoary^38^. Each gene cluster in the accessory genome was scored according to its apparent correlation to a predefined trait defined as Italian population, and Benjiamini-Hochberg (BH) P-value was calculated. Of the 9,085 accessory gene clusters, Scoary reported a total of 49 loci with a BH value under 0.05, and present in the 20% or more of Italian and the 30% or less of non-Italian isolates (Table 1). Loci clusters are located in separate fragments of the genomes, most of which exhibited homology to various genetic regions including phages, prophages and plasmid-associated genes originating from different bacterial species (*Salmonella enterica, E. coli* and *Shigella*). Thus, gene clusters have been divided in three groups: group 1 includes 7 contiguous loci belonging to a putative plasmid; group 2 includes 33 contiguous loci belonging to a large 42.9 kb prophage region; and group 3 includes the remaining 9 genes which are spread across the genome. These 49 loci are overrepresented in the STY-clade genomes (Supplementary Fig. S2). Particularly, only a single Italian isolate harbouring both group 1 and group 2 loci, and five UK isolates possessing a significant amount of group 2 loci are located outside the STY-clade. Finally, although clearly dominant in STY-clade, group 3 loci have been found quite frequently across the tree.

**Table 1:** Accessory genes overrepresented in Italian strains rated by Benjiamini Hochberg (BH) P-value.

Roary gene name	Prokka annotation	BH P-value	Italian isolates pos*	Italian isolates neg*	Non-Italian isolates pos**	Non-Italian isolates neg**	Gene details***
group_3215	Hypothetical protein	1,94E-48	70	108	15	1133	Phage BRO family/N-terminal domain protein
group_5738	Hypothetical protein	2,97E-47	70	108	17	1131	Phage lambda NC_001416: cell lysis protein/endopeptidase
group_7354	Hypothetical protein	3,54E-47	69	109	16	1132	Phege Clostr CDMH1 NC_024144: putative signalling/NTPase protein
group_7352	Hypothetical protein	3,54E-47	69	109	16	1132	Phage hypotetical protein
group_5725	Hypothetical protein	4,43E-47	70	108	18	1130	Phage Erwini phiEt88_NC_015295: DNA N-6-adenine-methyltransferase
group_2349	Hypothetical protein	4,43E-47	70	108	18	1130	Phage Entero SfI NC_027339: Ren protein
group_5737	Hypothetical protein	4,43E-47	70	108	18	1130	Phage Entero lambda NC_001416: Bor protein precursor
group_2253	Hypothetical protein	4,43E-47	70	108	18	1130	Phage Entero c_1 NC_019706: lysozyme
group_7353	Hypothetical protein	8,17E-47	69	109	17	1131	Phage Gifsy_1 NC_010392: bacteriophage antiterminator protein Q
group_7359	Hypothetical protein	3,28E-46	69	109	18	1130	Phage Entero 933 W NC_000924: hypothetical protein
group_3054	Hypothetical protein	4,28E-46	72	106	23	1125	Phage Shigel SfII NC_021857: hypothetical protein
group_4040	Hypothetical protein	1,98E-45	71	107	23	1125	Phage hypothetical protein
rusA_2	Crossover junction endodeoxyribonuclease	3,80E-45	70	108	22	1126	Phage Entero mEp237 NC_019704: Holliday junction resolvase RusA
group_7356	Hypothetical protein	5,10E-45	63	115	12	1136	Outer membrane protein assembly factor BamE
group_4041	Hypothetical protein	9,35E-45	70	108	23	1125	Phage Entero BP 4795_NC_004813: hypothetical protein
group_3216	Hypothetical protein	9,35E-45	70	108	23	1125	Phage Entero SfI NC_027339: replication protein P
group_3214	Hypothetical protein	9,35E-45	70	108	23	1125	Phage hypothetical protein
group_3213	Hypothetical protein	9,35E-45	70	108	23	1125	Phage hypothetical protein
group_1275	Hypothetical protein	9,35E-45	70	108	23	1125	Phage Salmon SEN34 NC_028699: replication protein O
group_4491	Hypothetical protein	9,35E-45	70	108	23	1125	Phage Rha protein
group_4493	Hypothetical protein	9,35E-45	70	108	23	1125	Phage Stx2 II NC_004914: hypothetical protein
group_7358	Hypothetical protein	9,35E-45	70	108	23	1125	Phage Entero 933 W NC_000924: host-nuclease inhibitor protein Gam
group_7351	Hypothetical protein	9,35E-45	70	108	23	1125	Phage Salmon ST64T NC_004348: holin protein
group_932	Hypothetical protein	9,35E-45	70	108	23	1125	Phage Entero phi80 NC_021190: CII decision making protein
kilR	Killing protein KilR	9,35E-45	70	108	23	1125	Phage Entero HK225 NC_019717: Kil protein
group_3075	Hypothetical protein	2,45E-44	134	44	233	915	Hypothetical protein
group_3217	Hypothetical protein	2,37E-43	69	109	24	1124	Phage Entero 933 W NC_000924: Bet protein
group_2348	Hypothetical protein	1,65E-42	63	115	16	1132	Predicted NTPase, NACHT family domain [Signal transduction mechanisms]/Ecoli
group_686	Hypothetical protein	3,66E-42	71	107	30	1118	Putative plasmid associated gene
group_2346	Hypothetical protein	4,12E-42	67	111	23	1125	Phage hypothetical protein
group_7349	Hypothetical protein	5,77E-42	68	110	25	1123	Putative plasmid associated gene
group_7350	Hypothetical protein	1,87E-41	68	110	26	1122	Putative plasmid associated gene
group_7348	Hypothetical protein	3,53E-40	66	112	25	1123	Putative plasmid associated gene
group_3117	Hypothetical protein	9,90E-39	51	127	6	1142	Genomic DNA
group_1265	Hypothetical protein	4,26E-35	70	108	45	1103	Putative plasmid associated gene
group_7650	Hypothetical protein	4,42E-35	52	126	12	1136	Genomic DNA
group_6567	Hypothetical protein	1,48E-33	60	118	28	1120	Genommic DNA
group_3072	Hypothetical protein	1,47E-32	111	67	194	954	Genomic DNA
group_7355	Hypothetical protein	2,21E-26	42	136	12	1136	Genomic DNA
prtR	Putative HTH-type transcriptional regulator	1,55E-25	41	137	12	1136	O antigen synthesis gene
group_7816	Hypothetical protein	3,86E-24	40	138	13	1135	Phage hypothetical protein
group_7817	Putative HTH-type transcriptional regulator	2,65E-23	39	139	13	1135	Phage Salmon ST160 NC_014900: C2 phage
rop	Regulatory protein rop	6,27E-20	79	99	140	1008	Putative plasmid associated gene
mbeC	Mobilization protein MbeC	2,84E-10	45	133	78	1070	Putative plasmid associated gene
group_48	Hypothetical protein	6,61E-04	44	134	141	1007	Genomic DNA
xerC_1	Tyrosine recombinase XerC	7,22E-04	80	98	334	814	Phage Shigel SfII_NC_021857: integrase
group_7044	Hypothetical protein	1,00E-03	79	99	333	815	Genomic DNA
group_4380	Hypothetical protein	1,48E-03	79	99	337	811	Phage protein flxA
sopE	Guanine nucleotide exchange factor SopE	4,38E-02	73	105	345	803	Phage G-nucleotide exchange factor SopE

*Number of Italian strains positive (pos) or negative (neg) for the observed gene.

**Number of non-Italian strains positive (pos) or negative (neg) for the observed gene.

***Gene details based on PHAST and on BLASTn against NCBI database.

### Characterization of the prophage region

**Figure 1:**
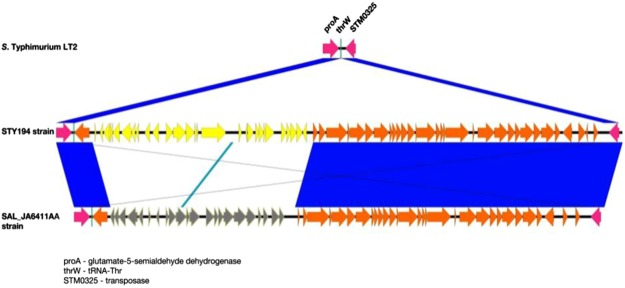
MVSTm prophage region alignment. Alignment of 42.9Kb prophage region of STY194 strain (in the middle) including 62 loci of which 33 (yellow) are that classified by Scoary^38^ as strongly associated with Italian MVSTm (group 2 in the text). At the top, S. Typhimurium LT2 showing the insertion of the prophage between the tRNA-thrW locus downstream of proA and the transposase STM0325. At the bottom, similar prophage region of UK strain SAL_JA6411AA with divergent loci coloured in grey. The full match of shared loci (orange and magenta arrows) is showed in blue.

A total of 10 prophages regions have been annotated by PHAST^39^ in the genome of the strain STY194, selected herein as reference genome for STY-clade. Of those 10 regions, 6 were intact, 3 were incomplete and one questionable. Among the intact regions, the one from nucleotide position 1,153,230 to 1,196,161 (42.9 Kb in total) includes 62 loci of which 33 have previously been classified by Scoary^38^ as strongly associated with Italian MVSTm (group 2, see above). The phage has been integrated downstream the *tRNA-thrW* gene homolog of *S*. Typhimurium LT2 (Fig. 1). A similar phage has been detected also in the UK strain SAL_JA6411AA (Fig. 1), belonging to the STY-clade but missing the group 2 loci. The phage in SAL_JA6411AA shows identical 3′-end but a divergent 5′-end sequence compared with STY194. The divergent part includes the 33 Italian associated loci of group 2 in STY194, substituted in SAL_JA6411AA by 22 different genes. In STY194, the 5′-half of the phage comprises genes involved in transcription and regulation, integration-recombination and cell division, prophage repression, cellular lysis and serum resistance. The conserved 3′-end of the pro-phage, which accounts for the 45.2% of the entire prophage genome, shows high similarity to the *Shigella flexneri* prophage SfII, and mainly encodes proteins involved in capsid formation and DNA packaging (head, tail, and terminase). Most of the 24 loci of this region were found in more than 75% of all genomes. The final portion of this phage genetic region shows homology to *Salmonella* phage SP_004 tail fiber assembly protein followed by *sopE*, a G-nucleotide exchange factor protein from SopEϕ. Genes encoding for these two proteins were shared by roughly the 35% of analysed genomes and *sopE* has been found to be negatively associated with North American origin (Fisher’s exact test; P < 0.0001).

### Phylogenomic reconstruction of the MVSTm strains

**Figure 2:**
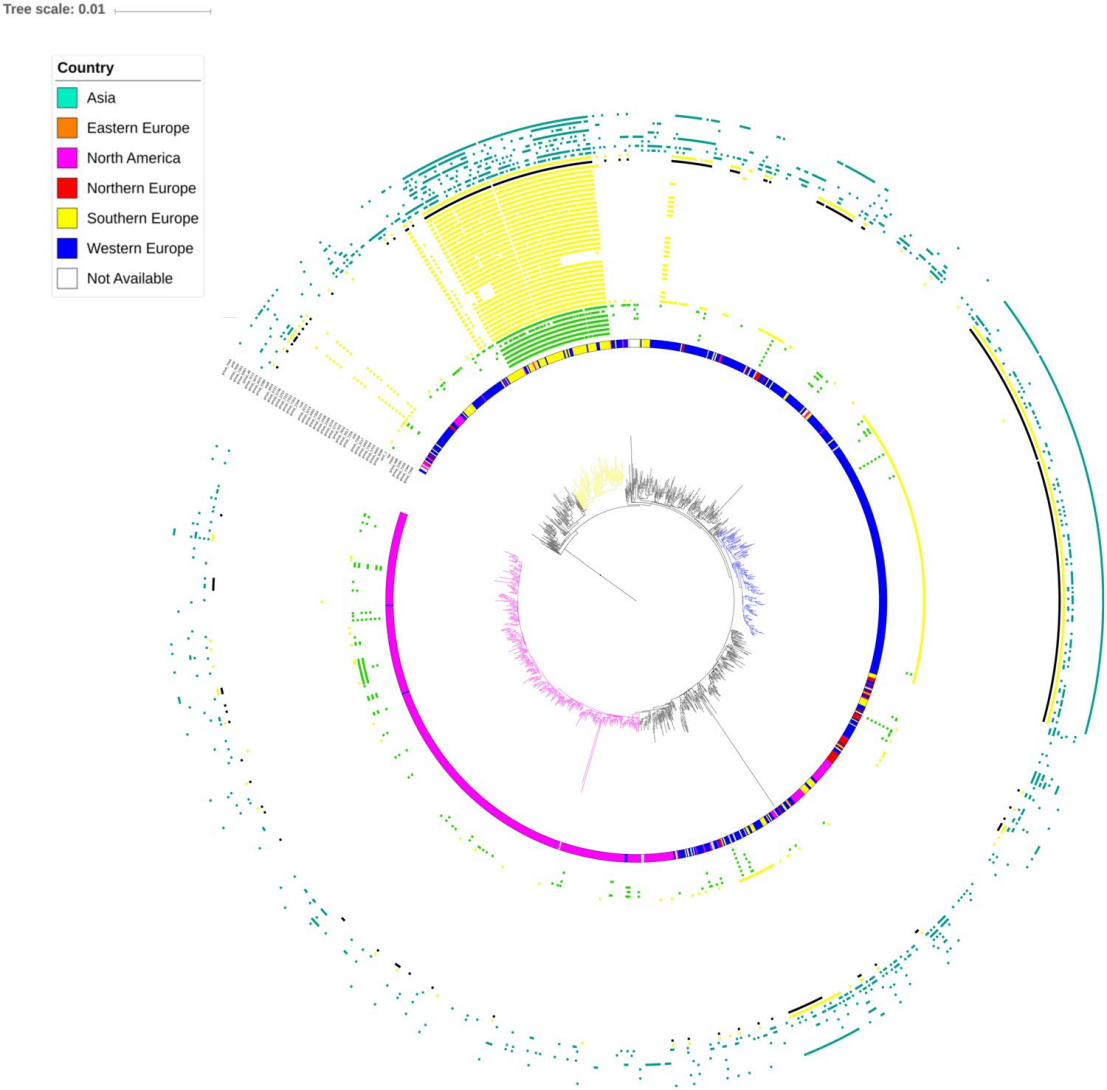
Core SNPs maximum likelihood tree. The maximum likelihood tree was inferred based on 11,278 core SNPs detected on 1,289 isolates. Figure 2 shows the tree pruned on clade II with coloured branches for subclade 10 (yellow), 41 (blue) and 61 (magenta). The internal circles indicate the originating geographical area of each isolates indicated by colours as in the legend. Externally, clusters of genes (detailed in Table 1) statistically associated with Italian strains divided by colours in plasmid-related contiguous loci (green); prophage related contiguous loci (yellow); and associated loci spread across the genome (light blue). The black hits are indicating *sopE* gene presence.

To better understand the genetic relationship of isolates characterized by different accessory genes profiles but gathered into a single goeBURST group showing up 2.5% of alleles differences, we performed a single nucleotide polymorphisms (SNPs) based phylogeny. All genome assemblies included in goeBURST^75^ (1,289) were mapped against the STY-clade reference genome using Snippy. Pairwise SNPs differences ranged between 0 to 1,793 with a median of 334 and a median percentage of bases aligned to the reference of the 98,43%. The maximum likelihood phylogeny and population structure were inferred based on 11,278 core SNPs. Two populations were identified corresponding to two major clades in the ML tree (Supplementary Fig. S3). Clade I is characterized by long branches and includes 25 MVSTm and one *S*. Typhimurium isolates of ST-19 mainly isolated in North American (~65%). More than half of the genomes exclusively included in clade I harboured from 10 to 32 pSLT-related genes. On the contrary, clade II is characterized by very short branches and includes 1,267 MVSTm (1,025) and *S*. Typhimurium (242) isolates mainly belonging to ST-34 (97,9%) and ASSuT genotype (66,3%), and collected in Europe (60%) and North America (38%) (Fig. 2). At 0.013 (nucleotide substitutions per site) distance from the root, clade II was divided in 61 subclades (containing at least two genomes) and 56 singletons. The subclades 10, 41 and 61, composed by 91, 195 and 474 genomes, respectively, account for ~60% of the genomes in clade II and are significantly associated to the origin of the isolates. Subclade 10 (which contains the reference genome) is significantly associated with Italian origin (Fisher’s exact test; P < 0.0001) and includes ~50% of the Italian STY-isolates available in the dataset and 5 additional Italian MVSTm isolates that were collected prior to 2012. In subclade 10, pairwise distance was from 0 to a max of 368 (Supplementary Fig. S4). In comparison to the accessory genome clustering, subclade 10 contains 85/91 isolates belonging to STY-clade and all but 5 possess the *sopE*-containing phage. The distribution of the loci overrepresented in the Italian isolates on the core SNP tree, as shown in Fig. 2, indicate a clear association with subclade 10, particularly for the group 1 and 2 loci. Subclade 41 is significantly associated with Western Europe origin (UK and Ireland; Fisher’s exact test; P < 0.0001) while subclade 61 is significantly associated with North American origin (Fisher’s exact test; P < 0.0001), comprising 88% of the North American isolates included in the study.

### Bio-markers distribution

**Figure 3:**
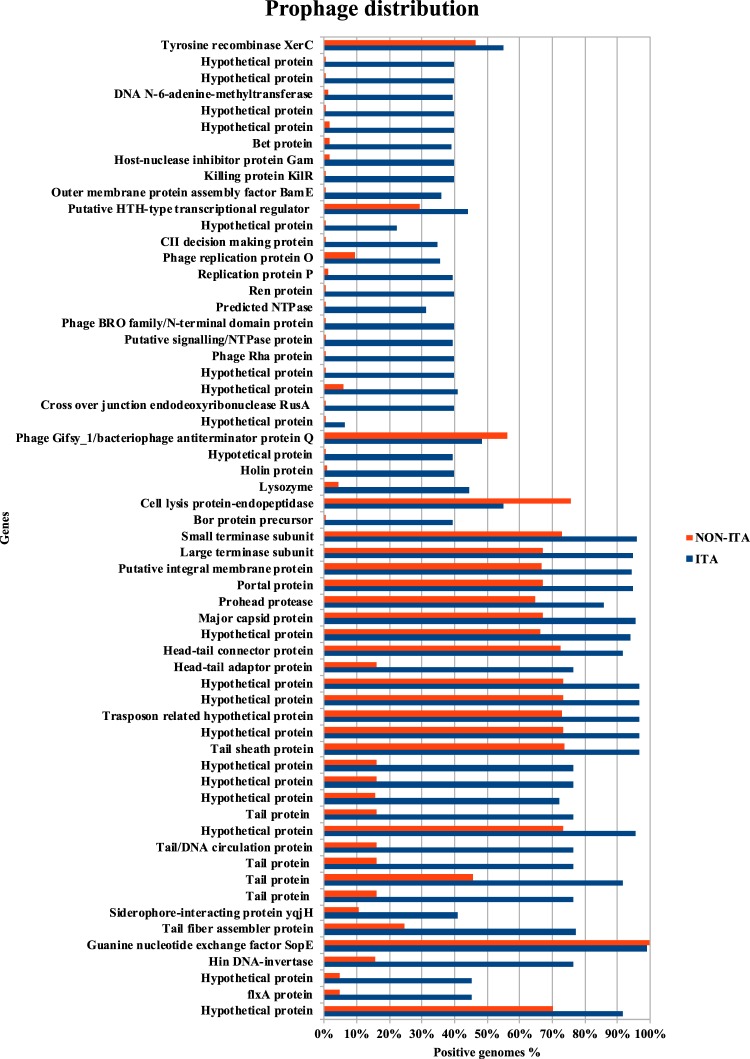
SopEϕ loci distribution between Italian and non-Italian isolates. The percentage of genomes with positive match for each locus included in *sopE*-containing prophage is reported in the chart according to Italian (blue) and non-Italian (orange) origin. Gene details for each locus is indicated on the left as reported by PHAST annotation.

We investigated the distribution of SopEϕ containing the genes overrepresented in Italian MVSTm isolates in an extended *Salmonella enterica* dataset consisting of 8,787 genomes including additional 4,215 publicly available *S*. Typhimurium and its monophasic variant (Supplementary Table S4). The new dataset contains a total of 5,660 *S*. Typhimurium, 1,084 MVSTm, 1,518 Enteritidis and 525 others *Salmonella* serovars. All the CDSs of SopEϕ from STY194 were screened against this dataset. The presence/absence of each locus according to origin of the strain (Italian vs non-Italian) is summarized in Fig. 3, which shows a clear overrepresentation of the 5′-half of the SopEϕ in samples of Italian origin. We further divided the samples in four categories, according to percentage of positive matches of SopEϕ loci (Table 2), considering ≥90% of positive matches as positive for SopEϕ (group A). Roughly 42% (66) of the Italian MVSTm isolates over the whole dataset were included in group A along with 11 isolates from the Western European area (UK, Ireland, Luxembourg) and 2 from North America (US). Strains in group A, 74 of which are members of the Italian associated subclade 10 of goeBURST^75^ cluster described above, were isolated from human and swine and were collected over 10 years (2007–2017). Approximately 5% of the samples in the dataset were positive for at least 50% of the SopEϕ loci (group B), 95% of which (337) were either serovars *S*. Typhimurium (25%) or MVSTm (71%) and the remains belong to serovar Derby. The majority (285) of *S*. Typhimurium/MVSTm genomes included in group B has been collected from human samples in European countries, particularly UK (187), while a limited number (35) was from American isolates collected from different sources. Roughly 90% of the genomes belonging to the UK associated subclade 41 of goeBURST^75^ cluster were classified as group B. The remaining 8,356 genomes show only limited positive matches (<50%) for SopEϕ loci. Around 40% of Italian MVSTm isolates were included in group C (≥30% of loci) along with other 4,759 *S*. Typhimurium/MVSTm genomes collected over a century (i.e. collection timeframe ranges between 1917 and 2017) across five continents. In addition, 95% of the genomes belonging to the North America associated subclade 61 of goeBURST^75^ cluster were classified in group C. The remaining 1,503 *S*. Typhimurium/MVSTm genomes, including 4 Italian MVSTm isolates clustered in subclade 10, did show less than 30% or no positive matches (group D).

**Table 2:** Categorization of 8,787 *Salmonella* genomes based on the distribution of biogeographical markers.

Group	% of positive matches of SopEϕ loci	Number of genomes	% of the total dataset of 8,787 genomes
A	≥90% (equal to SopEϕ positive)	79	1%
B	≥50% < 90%	352	4%
C	≥30% < 50%	4,905	56%
D	<30% or no matches	3,451	39%

These data suggest that this SopEϕ is rather bound up to subclade 10 of goeBURST^75^ cluster. We further verified that 529 out 4,217 *S*. Typhimurium/MVSTm genomes from the novel dataset belong to goeBURST^75^, including genomes classified as group A (3) and B (22). However, only genomes from group A, collected in Italy from 2007 to 2009, clustered in subclade 10 (Supplementary Fig. S5), supporting the hypothesis that this feature is characteristic of this subclade.

## Discussion

The importance of *S*. 1,4,[5],12:i:- (MVSTm) arose when it climbed up the charts of *Salmonella* serovar responsible of food-borne outbreaks worldwide. In particular, in Europe the joint report on zoonosis monitoring by EFSA and ECDC described *S*. 1,4,[5],12:i:- as the third serovar among *Salmonella* already in 2013^6^. Swine appears to be the main reservoir of this peculiar *S. enterica* serovar. However, the monomorphic nature of MVSTm has been an obstacle for identifying the relative importance of other animal species as sources of human infections^40^.

Several studies have been conducted in recent years to elucidate the phylogenetic relationship, transmission dynamics as well as the virulence and resistance key determinants of epidemics MVSTm^11,12,33,41–46^. Genomic analyses have suggested the emerging of multiple independent clones in the United States and Europe^4^. Specifically, three distinct epidemics have driven the microevolution of MVSTm across the globe resulting in the expansion of different clones which tend to be dominant in specific geographical locations^41^. Recent data on *Salmonella* serovar Cerro suggested that several genomic markers associated to geographically segregated phylogroups may contribute to the ability of *Salmonella* to rapidly diverge and adapt to a specific niche^47^. Therefore, geographical segregation can play an important role in the microevolution of emerging clones, leaving enough genetic signal in the population which can contribute to improve source attribution of clinical MVSTm infections. In the present study, we tested this hypothesis by focusing on identifying biogeographical markers in MVSTm genomes of a geographical and temporal related set of isolates obtained in Italy between 2012 and 2014. To mine phylogenetic related isolates from different part of the world, we applied a naïve approach by comparing the 148 Italian isolates with a large set (>4,000) of representative publicly available genomes of several *S. enterica* serovars using the gene-by-gene methodology. Thus, we identified ~1,300 *S*. Typhimurium and MVSTm from a broad geographical area collected in almost 20 years from several sources showing up to 2.5% allele diversity with most of the Italian isolates. By integrating phylodynamics with genome-wide association analysis, we have shown that within this peculiar population of very similar *S*. Typhimurium/MVSTm isolates, the expansion of genotypes in a specific geographical region is facilitated by the acquisition of unique accessory genetic markers. Phylogenetic reconstruction revealed that isolates from the same geographical origin form several highly supported monophyletic groups, providing discrete evidence of the phylogeographical structure of this population. Isolates from human and from swine related sources clustered in these groups indicating that humans are exposed to the same genotypes circulating among pigs. The presence of most of the Italian isolates collected over 7 years of sampling within a single monophyletic clade characterized by specific repertoire of plasmid- and phage-related loci supports the hypothesis that this genotype endured a substantial genetic differentiation. Among prophage elements, we found *sopE* gene, a virulence factor recently described in European strains^41^, enriched in genomes from subclades 10 and subclades 41, significantly associated with Italian and UK origin, respectively. This is consistent with findings by Petrovska and colleagues suggesting an increase of *sopE* gene frequency since 2007 in monophasic epidemic isolates from UK and Italy^41^; increase confirmed by the overrepresentation of *sopE* gene in the European MVSTm collected after 2010 (350/727; ~48%) analysed in this study. Although our data show a negative association of this gene with isolates from North America, a recent study by Elnekave and colleagues^42^ reported the presence of *sopE* gene in US isolates from swine samples collected during 2014–2016. The fact that MVSTm isolates harbouring *sopE* gene have been collected in Europe for several years before may raise the question of whether *sopE* positive isolates in US are most likely originating from European strains circulating in swine production chain. Further studies are needed to elucidate with higher resolution the genetic relationship of *sopE* positive isolates on a more representative set of US and European swine-related MVSTm strains.

Noteworthy, whereas prophage virulence gene *sopE* was mostly located in strains from Italian and Western Europe phylogroups in ST-34 clade II, *spvC* and *spvB* and other virulence markers of pSLT plasmid with reported contribution to pathogenicity of *S*. Typhimurium^36,48^ were located in strains from clade I belonging to ST-19. The presence of these virulence genes exclusively in ST-19 strains provided evidence that these strains most likely originated from *S*. Typhimurium ancestors distinct from that of the European clone. This is consistent with results of García studies^13,49,50^ where the presence of *spvC* gene and virulence-plasmids genes is reported only in MVSTm ST-19 strains. Although *spvC* positive ST-19 MVSTm strains have been described as similar to the hepta-resistant Spanish clone, antimicrobial resistance (AR) genes were found in only 4 out of 15 of the virulence-harbouring isolates of our dataset. In the current study, we showed that the most common AR profile for the majority of *Salmonella* Typhimurium/MVSTm strains isolated from humans, animals, environment and animal foodstuffs included in ST-34 clade II predict ASSuT genotype. The wide diffusion of multiple genotypes of R-type ASSuT MVSTm in European countries as well as in North America and Asia constitute a growing risk that can be associated to increased hospitalization, development of a bloodstream infection, or treatment inefficacy in patients^51^. However, the local expansion of specific clones can also result in the loss of AR genes, as we observed in the UK-associated subclone 41. The loss of ASSuT or ACGSSuTTp genotypes emerged towards the terminal branches of the subclade 41 tree, populated exclusively by isolates harbouring a single tetracycline resistance gene. Since within this subclade time of isolation of mono-resistant genotypes is subsequent to that of multi-resistant genotypes, we presume that the dynamic genome plasticity of *S*. Typhimurium/MVSTm serovars may lead to the formation and successful expansion of clones suffering the loss of particular adaptive traits.

Petrovska and colleagues^41^ have investigated the microevolution of MVSTm clones responsible for recent UK epidemic ways (from 2005 to 2012). The authors discovered that monophasic epidemic clones circulating in UK and Italy are characterized by the acquisition of multiple novel genes, including a *sopE*-containing prophage mTmV, and formed a single clade with remarkable genetic variation from North American and Spanish epidemics clones. They identified three distinct subclades one of which (i.e. subclade C) being preferentially associated with Italian livestock production. In our study, four of the Italian isolates collected up to 2010 included in the study of Petrovska and colleagues^41^ belong to the Italian associated subclade 10 as shown in Fig. 2, together with ~57% of the Italian STY isolates analysed in this study and collected from 2012 to 2014. Particularly, the subclade 10 represents a significant expansion of the *sopE* positive monophyletic group within subclade C as described by Petrovska and colleagues^41^. Similarly, the UK associated subclade 41, as shown in Fig. 2 herein, represents the recent expansion (>90% of the samples within subclade 41 were collected in UK or Ireland after 2014) of *sopE* positive monophyletic group within subclade A described by Petrovska and colleagues^41^. The ongoing clonal expansion of these *sopE* positive MVSTm subpopulations shows that the acquisition of this gene has conferred a clear competitive advantage in the ongoing European MVSTm epidemics. As previously suggested^41^, the acquisition of *sopE* has happened in multiple independent events. This theory is confirmed by the gene contents of the *sopE*-containing prophage. Indeed, in the UK associated subclade 41, *sopE* is located at the 3′-end of a prophage mTmV as previous described^41^, while in the Italian subclade 10 the prophage containing *sopE* shared only half of the mTmV genes. This novel prophage, mTmV2, contains the majority of the loci overrepresented in Italian isolates and is characteristic of subclade 10 as confirmed by investigating the prophage presence on a novel dataset of thousands of *S*. Typhimurium and its monophasic variant genomes. Results from this analysis underlined that no strains outside the goeBURST^75^ population contains mTmV2 prophage and that the addition of new genomes doesn’t change the topology of the tree, which shows mTmV2 positive genomes, classified as group A, clustering within subclade 10.

The gain and loss of mobile genetic elements may “unlock the secrets” for the optimization of infection-control strategies and effective containment of emergent pathogens, as was already discussed in a recent study on the transmission dynamics of *Enterococcus faecium*^52^. Therefore, we can conclude that investigating on the presence of particular genetic elements, such as mTmV2 prophage, can contribute in enhancing the ability in tracking the dissemination of specific clones of MVSTm in ongoing epidemics.

## Methods

### Bacterial strains, genome sequencing and assembling

A total of 148 *Salmonella enterica* serovar Typhimurium variant 4,[5],12:i:- (MVSTm) have been collected from different Italian regions between 2012 and 2014 during a surveillance study. For the aim of this study, this dataset has been named STY. Pig faecal samples (11), pork carcass isolates (23) and pork meat at retail isolates (27) were obtained from the Italian National Reference Laboratory for *Salmonella* (NRL *Salmonella*, Istituto Zooprofilattico Sperimentale delle Venezie, Legnaro, Italy), while isolates from humans with gastroenteritis (82) were obtained from the National Institute of Health (Instituto Superiore della Sanitá, Roma). Genomic DNA of the 148 STY isolates was extracted and purified using the HWD DNA minikit (QIAGEN) according to the manufacturer’s instruction. Index-tagged paired-end Illumina sequencing libraries were prepared using NexteraXT library preparation kit and whole genome sequencing was performed on Illumina MiSeq platform generating tagged 250 bp paired-end reads. The paired-end raw reads were assembled using the INNUca pipeline^53^ (https://github.com/theInnuendoProject/INNUca), which consists of several modules and QA/QC steps. In brief, INNUca starts by calculating if the sample raw data fulfil the expected coverage (min 15x). After subjecting reads to quality analysis using FastQC, and cleaning with Trimmomatic^54^, INNUca proceeds to *de novo* draft genome assembly with SPAdes^55^ v3.11 checking assembly depth of coverage (min 30x). Finally, Pilon^56^ improves the draft genome by correcting bases, fixing misassembles and filling gaps; prior species confirmation and seven genes MLST Sequence Type (ST) is assigned with mlst software (https://github.com/tseemann/mlst).

### Reference genomic collection for investigating *Salmonella* population structure

To assess the population structure of monophasic *Salmonella* Typhimurium isolates in comparison to a *Salmonella enterica* reference collection, 4,312 publicly available draft or complete genome assemblies along with available metadata have been downloaded from public repositories (i.e. EnteroBase - https://enterobase.warwick.ac.uk/, National Center for Biotechnology Information NCBI - https://www.ncbi.nlm.nih.gov/ and The European Bioinformatics Institute EMBL-EBI - https://www.ebi.ac.uk/; accessed April 2017). The reference collection includes 1,465 *Salmonella enterica* ser. Enteritidis, 1,425 ser. Typhimurium, 985 MVSTm, and 437 of other frequently isolated serovars in Europe according to EFSA and ECDC joint summary report on trends and sources of zoonoses, zoonotic agents and food-borne outbreaks (EFSA/ECDC, 2016). All available assemblies for MVSTm have been chosen. For each of the other serovars, genomes have been selected to maintain the same proportions of genetic diversity representatives of all the diversity revealed by rMLST^18^ as existing in EnteroBase at the date of collection (April 2017) and to design a suitable and accurate cgMLST schema which was also able to validate if the genomes serotypes were correctly annotated in the available metadata.

### Core genome MLST (cgMLST) allele calling and cluster analysis

Population structure analysis of *Salmonella* genomes have been performed using cgMLST methodology^57^. Schema curation, validation and allele calling have been carried out using the chewBBACA^58^ suite as described by Rossi and colleagues^59^ (https://github.com/theInnendoProject/chewBBACA_schemas). Briefly, the wgMLST schema V2 from EnteroBase, including 21,064 loci, have been downloaded and curated using *chewBBACA* suite resulting in a total of 8,558 loci. The core genome MLST profile, defined as the loci presence in at least the 99% of the samples, has been then extracted using *chewBBACA ExtractCgMLST* consisting of 3,255 loci. Genomes with more than 2% of missing loci have been excluded.

The global optimal eBUSRT algorithm (goeBURST)^60^ implemented in Phyloviz^31^ 2.0 has been used to identify cluster membership of cgMLST profiles of *Salmonella* strains at different thresholds of allelic differences. Neighbourhood Adjusted Wallace Coefficient (nAWC)^61^ was calculated to assess cluster consolidation dynamics. nAWC examines the congruence of partitions between adjacent similarity thresholds used for cluster definition (https://github.com/theInnuendoProject/nAWC). nAWC identifies areas in in which distance thresholds produce robust clusters reflecting basic units in *Salmonella* overall population structure^61^. In addition, we also evaluate concordance between partitions obtained at different goeBURST cut-off and serotyping with Adjusted Wallace Coefficient^62^ (AWC).

### Genome annotation, pangenome analyses and Genome-Wide Association Study (GWAS)

Pangenome analysis was performed on all genomes belonging to a selected goeBURST group, henceforth referred to as goeBURST^75^, previously defined for showing up to 2.5% of allele differences and for including 136 newly sequenced STY MVSTm isolates. A set of outliers composed of 38 *S*. Typhimurium/MVSTm genomes, including additional 27 Italian MVSTm outbreak-related strains, was also included in the analysis in order to gain further genome diversity and comparability. *Salmonella* genomes were annotated with Prokka^63^ (https://github.com/tseemann/prokka) and the produced GFF3 files were used to generate the pan-genome matrix with Roary^32^ (https://github.com/sanger-pathogens/Roary) using default parameters. The neighbourhood joining tree based on the binary matrix of presence and absence of accessory genes calculated by Roary^32^ was visualized on iTOL^64^ v3 (https://itol.embl.de) along with relevant metadata. A GWAS was performed based on Roary^32^ results using Scoary^38^ (https://github.com/AdmiralenOla/Scoary) v1.6.16 with default parameters. Patterns of genes were reported as significantly associated to geographical origin (e.g. Italy) if they attained a Benjiamini-Hochberg-corrected P-value less than 0.05 and were present in at least the 20% of the selected isolates (e.g. Italian) and absent in at least the 70% of the rest of the dataset (e.g. non-Italian isolates). The synteny of the associated loci was visually assessed using Artemis^65^ annotation tool on a selection of STY isolates and further manually annotated. If the associated genes were annotated as hypothetical protein the gene was manually curated by searching homologs sequencing in non-redundant (nr) NCBI protein sequences collection using blast+^66^ v2.7.1 (https://blast.ncbi.nlm.nih.gov/). We investigated presence and distribution of the geographic associated loci on an extended *Salmonella enterica* dataset inclusive of 8,787 genomes (Supplementary Table S4). This dataset included all genomes of the reference collection along with a novel curated set of 4,217 publicly available *S*. Typhimurium genomes: all public genomes not already included in the reference collection, deposed in SRA or ENA as *S*. Typhimurium or its monophasic variant for which country of isolation was available and of which assembly met minimum quality criteria (i.e. genome assembly length ranging 4.5–5.5 Mb). Presence/absence of the associated loci were performed using chewBBACA workflow^58^. Briefly, a schema composed by the associated loci were constructed and used for performing allele calling on all 8,787 genomes. The locus was marked as present if an allele was called or if it was annotated as “non-informative paralogous hit (NIPH/NIPHEM)” or if was detected on the tip of the query genome contigs^58^ (for details https://github.com/B-UMMI/chewBBACA/wiki/2.-Allele-Calling).

### In silico typing

Antibiotic resistance and plasmids prediction was performed with ABRicate pipeline (https://github.com/tseemann/abricate) using ResFinder^37^ and PladmidFinder^35^ as reference database. The typical AR profile of *S*. 1,4,[5],12:i:- “European clone” was defined as the simultaneous presence of *bla*_*TEM-1*_, *strA* (and its synonymous *aph(3“)-Ib*), *strB* (and its synonymous *aph(6)-Id*), *sul1*, *sul2* and *tet(B)* genes (R-type ASSuT)^12^. In addition, when *cmlA1*, *aac(3)-IV* and *dfrA12* genes were detected in that isolates harbouring ASSuT related genes they were predicted as resistance type ASSuTCGTp, a specific pattern associated to S. 4,[5],12:i:- from the “Spanish clone”^13^.

The presence of pSLT-genes encoding virulence factor in a 94-kb plasmid (AE006471) from *S*. Typhimurium LT2 was investigated using blastn implemented in blast+^66^ v2.7.1. The PHAge Search Tool (PHAST)^39^ was used to identify the positions of putative phage elements. For PHAST analysis, genomes have been annotated using RAST annotation server^67^. Hence, the annotated GBK file was uploaded to the public PHAST web server (http://phast.wishartlab.com/)^39^.

### Single-nucleotide polymorphism (SNP) analysis

To establish the phylogenetic relationship between closely related strains based on the goeBURST clustering, SNP analysis have been performed with Snippy v3.2 pipeline, using the assembled genomes as input files (https://github.com/tseemann/snippy). As reference, the best assembled draft genome (based on N-50 values and coverage) harbouring the largest set of geographical associated genes has been selected within the goeBURST^75^ cluster. A core alignment of all the conserved nucleotide variant sites present in all genomes was used to build a maximum-likelihood tree using IQ-tree^34^ with a gamma correction for site rate variation using 1,000 bootstrap replicates to support the nodes. hierBAPS^68^ was used for clustering the samples based on the core SNPs alignment up to second level.

## Electronic supplementary material

Supplementary Tables

Supplementary Figures

## Data Availability

Genome assemblies are accessible at the European Nucleotide Archive (https://www.ebi.ac.uk/ena) under the project accession number: PRJEB23875.
