# Supplementary material for: Genome-wide identification of geographical segregated genetic markers in Salmonella enterica serovar Typhimurium variant 4,[5],12:i:-
Source: Sci Rep. 2018 Oct 15;8:15251. doi: 10.1038/s41598-018-33266-5 (PMC6189080; doi:10.1038/s41598-018-33266-5)
Supplement: Supplementary file 2 — Supplementary Figures [file 41598_2018_33266_MOESM2_ESM.pdf]

Manuscript title: Genome-wide identification of geographical segregated genetic markers in *Salmonella enterica* serovar Typhimurium variant 4,[5],12:i:-

Authors: Federica Palma, Gerardo Manfreda, Mickael Silva, Antonio Parisi, Dillon O. R. Barker, Eduardo N. Taboada, Frédérique Pasquali, Mirko Rossi

Supplementary Figure S1: Minimum Spanning Tree of 1,326 genomes.

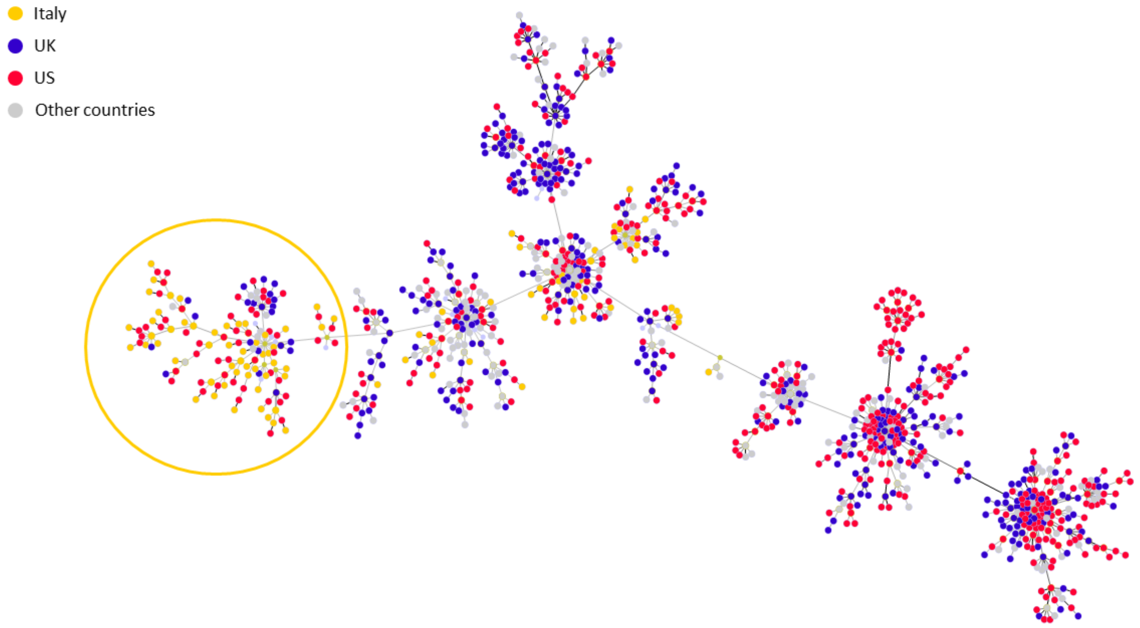

Minimum Spanning Tree based on a 3,591 loci cgMLST schema calculated using Phyloviz 2.0. The country of origin of the strains have been visualized on the tree by colours (Italian cluster, yellow circle).

Supplementary Figure S2: Distance tree based on accessory genes of 1,326 genomes.

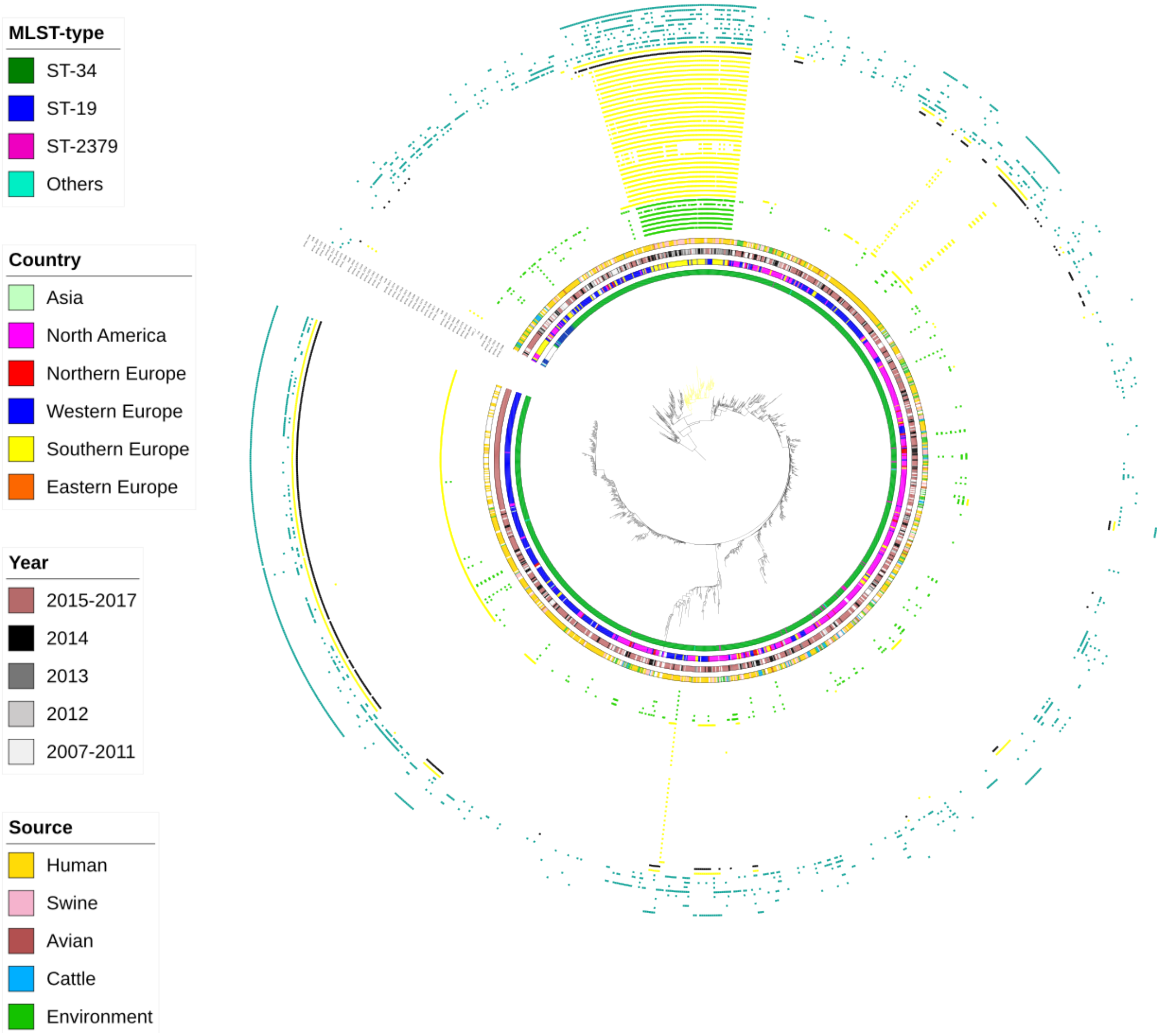

Distance tree inferred based on binary data of presence/absence of accessory gene of 1,326 S. Typhimurium/ MVSTm strains included in the study. From the inside out, MLST-types, originating country, year and source of isolation are indicated on the tree by colour as reported in the legend. Externally, clusters of genes statistically associated with Italian strains are labelled as from pangenome analysis and divided by colours in plasmid-related contiguous loci (green); prophage related contiguous loci (yellow); and associated loci spread across the genome (light blue). The black hits are indicating *sopE* gene presence while missing data are in white. The yellow coloured branch is indicating STY-clade.

Supplementary Figure S3: cgSNPs maximum likelihood phylogeny of 1,289 genomes.

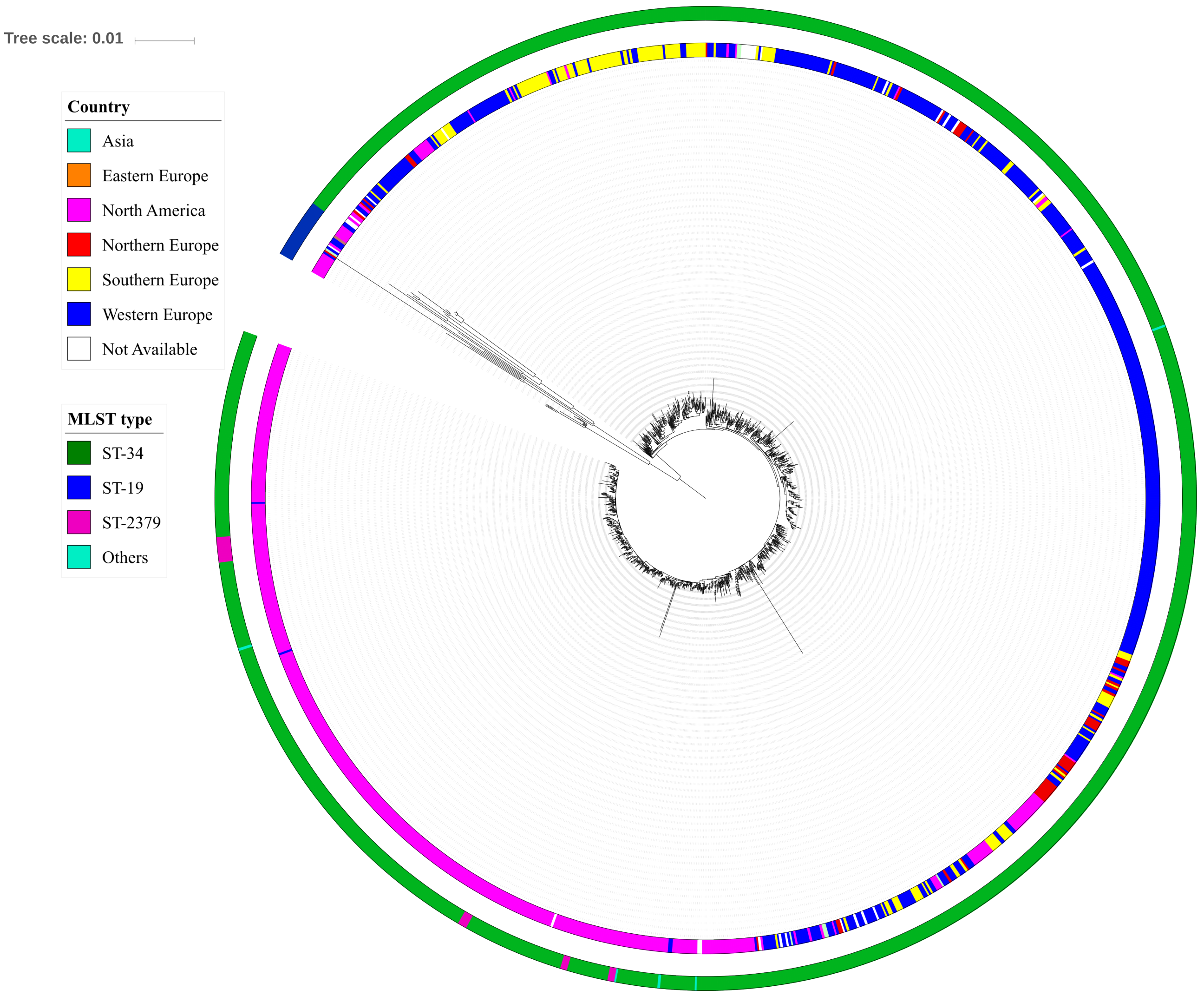

Maximum likelihood inferred based on 11,278 core genome SNPs. The tree shows the *S.* Typhimurium/ MVSTm population forming two major clades. The long branch indicates clade I including ST-19 strains (blue) mainly from North America. On the contrary, clade II mainly includes ST-34 strains (external circle). Countries of origin are indicated in the internal circle by colours as reported in the legend.

Supplementary Figure S4: Pairwise distance of core SNPs from subclade 10.

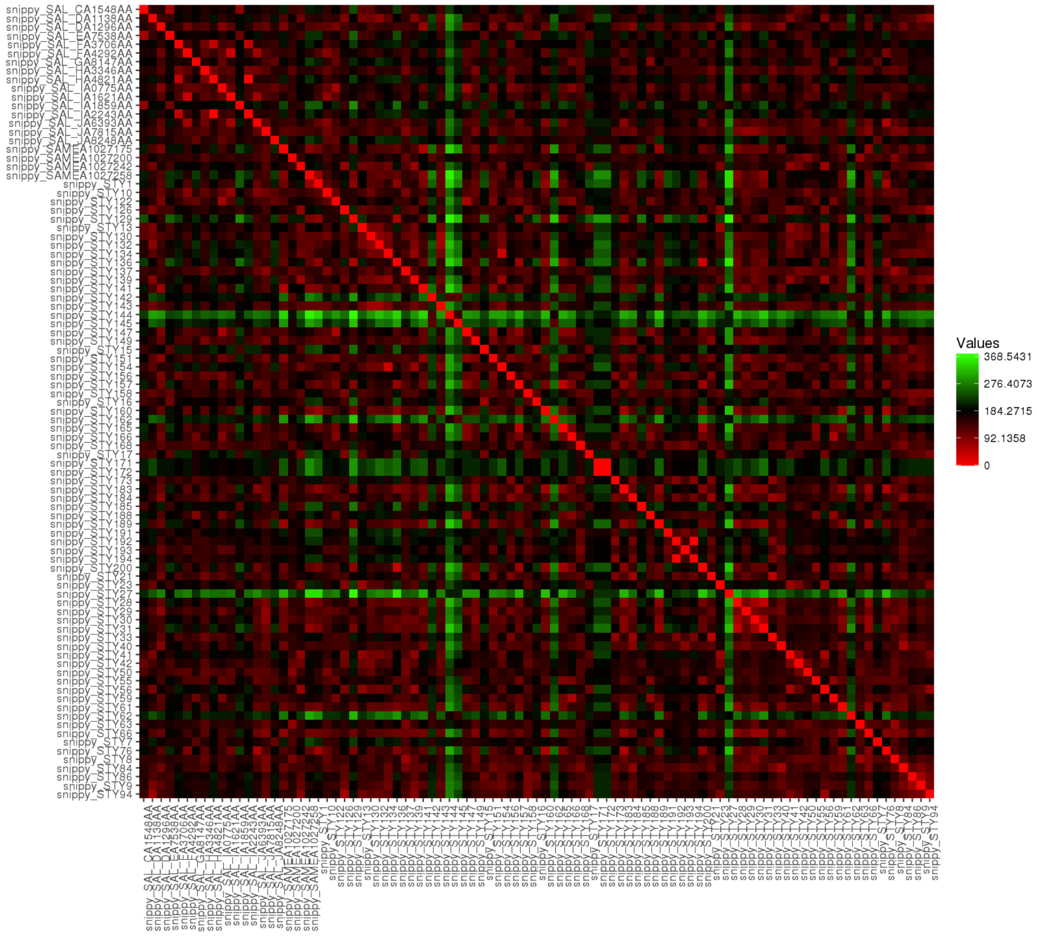

Pairwise SNPs distance matrix of the core genome SNPs alignment of strains from subclade 10. The pairwise distance ranges from 0 to a max of 368. SNPs distance was calculated with snps-dists ([https://github.com/tseemann/snp-dists)](https://github.com/tseemann/snp-dists) and visualized on http://www.heatmapper.ca.

Supplementary Figure S5: cgSNPs ML phylogeny of 1,818 goeBURST[75] genomes.

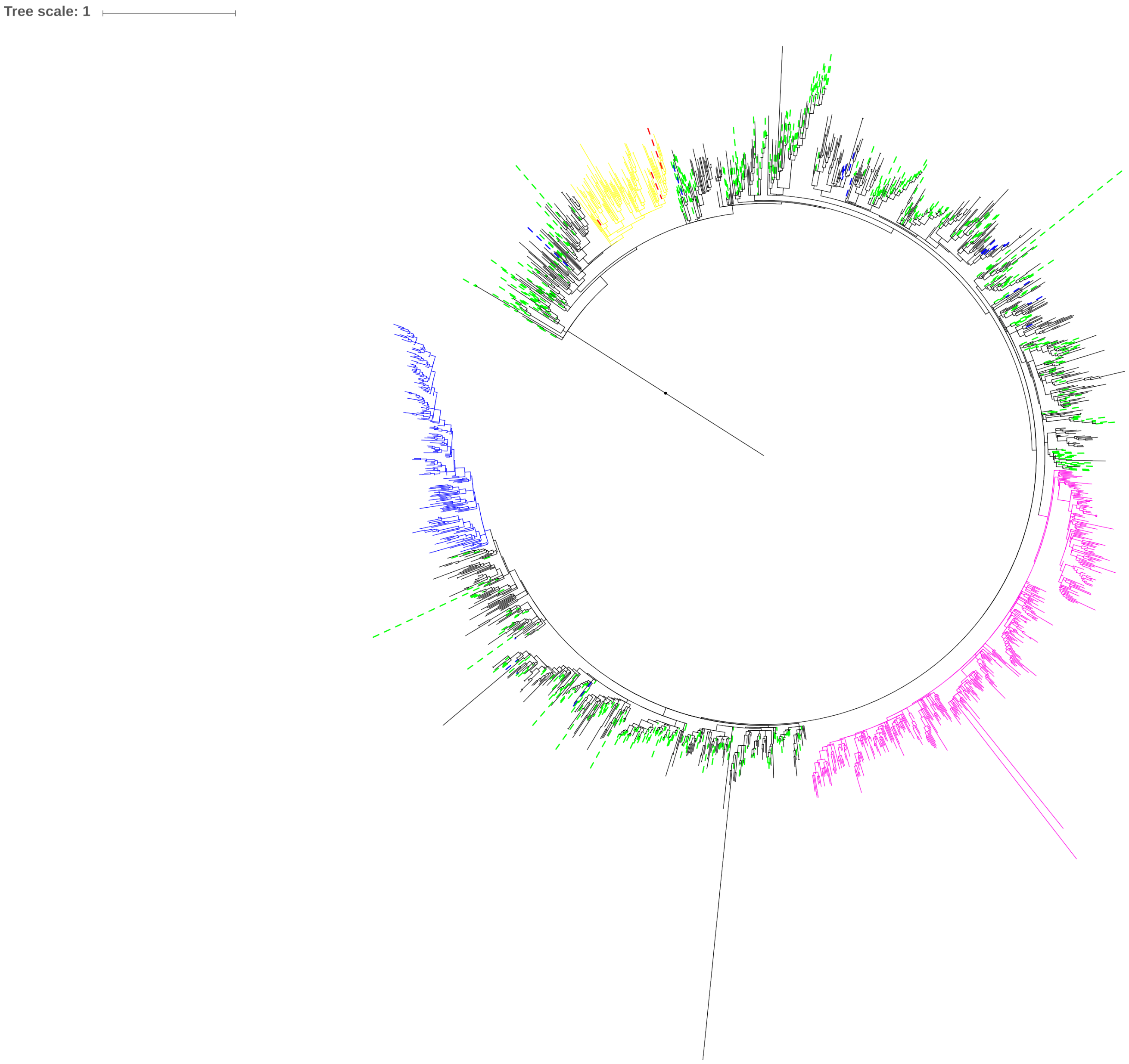

Maximum likelihood tree based on core genome SNPs alignment of 1,818 genomes from the new goeBURST[75] cluster including additional 529 *S.* Typhimurium/ MVSTm genomes with up to 2.5% of allele differences. The tree has been pruned on clade including ST-34 strains and shows similar clustering to the cgSNPs tree reported in Figure 2. The yellow, blue and magenta coloured branches indicate the previously identified Italy, Western Europe and North America associated subclade 10, 41 and 61. Additional genomes classified as group A, clustering within subclade 10, have been indicated with red dashed lines. Genomes classified as group B have been indicated with blue dashed lines whereas genomes belonging to group C and D have been indicated with green dashed lines.
